# Finite-Sample Bounds on the Accuracy of Plug-In Estimators of Fisher Information

**DOI:** 10.3390/e23050545

**Published:** 2021-04-28

**Authors:** Wei Cao, Alex Dytso, Michael Fauß, H. Vincent Poor

**Affiliations:** 1 National Key Lab of Science and Technology on Communications, University of Electronic Science and Technology of China, Chengdu 611731, China; clarissa.cao@hotmail.com; 2 Department of Electrical and Computer Engineering, New Jersey Institute of Technology, Newark, NJ 07102, USA; 3 Department of Electrical and Computer Engineering, Princeton University, Princeton, NJ 08544, USA; mfauss@princeton.edu (M.F.); poor@princeton.edu (H.V.P.)

**Keywords:** nonparametric estimation, Fisher information, MMSE, kernel estimation

## Abstract

Finite-sample bounds on the accuracy of Bhattacharya’s plug-in estimator for Fisher information are derived. These bounds are further improved by introducing a clipping step that allows for better control over the score function. This leads to superior upper bounds on the rates of convergence, albeit under slightly different regularity conditions. The performance bounds on both estimators are evaluated for the practically relevant case of a random variable contaminated by Gaussian noise. Moreover, using Brown’s identity, two corresponding estimators of the minimum mean-square error are proposed.

## 1. Introduction

This work considers the problem of estimating the Fisher information for the location of a univariate probability density function (PDF) *f* based on *n* random samples $Y_{1},\ldots ,Y_{n}$ independently drawn from *f*. To clarify, the Fisher information of a differentiable density function *f* is given by

(1) $$I\left(f\right)=\int_{\left\{f(t)>0\right\}}\frac{{\left(f^{\prime }\left(t\right)\right)}^{2}}{f(t)}dt,$$

where $f^{\prime }$ is the derivative of *f*. For the remainder of the paper, it is assumed that {f(t)>0}=R, but an extension to the general case is not difficult. The paper considers plug-in estimators based on kernel density estimates of *f*. That is, the Fisher information is estimated by plugging a kernel density estimate of *f* into the right-hand side of (1).

Estimation of the Fisher information in (1) via a plug-in estimator based on kernel density estimates was first considered by Bhattacharya in [1]. Bhattacharya showed that, under mild conditions on *f*, the plug-in estimator is consistent for a large class of kernels, and he provided bounds on its accuracy in the large (asymptotic) sample regime. These bounds were later revised and improved by Dmitriev and Tarasenko in [2]. However, to the best of our knowledge, no finite-sample regime bounds on the accuracy of Bhattacharya’s estimator can be found in the literature. The paper aims at closing this gap.

Bounds on the accuracy of plug-in estimators rely on bounds on the accuracy of the underlying density estimators. For kernel-based density estimators, such bounds have received considerable attention in the literature. For example, Schuster [3] showed that, under mild regularity conditions, the estimation error for higher-order derivatives can be controlled by the estimation error for the corresponding cumulative distribution function (CDF). The interested reader is referred to [4,5,6,7,8] and the references therein. In this paper, as a preliminary result for the analysis of Bhattacharya’s estimator, the bounds in [3] are further tightened by replacing some suboptimal constants with the optimal ones.

A problem that arises in the performance analysis of plug-in estimators for Fisher information is that the score function of the estimated density, that is, the ratio of the derivative of the PDF and the PDF itself, is hard to bound, especially in the tails. Bhattacharya worked around this problem by truncating the integration range in (1), thus avoiding evaluation of the estimated score function on these critical regions. However, in order for the estimator to stay consistent, this truncation has to be done rather aggressively so that the error introduced by ignoring the tails can outweigh the approximation error introduced by the density estimate. In this paper, we propose a simple remedy that allows for a much less aggressive truncation of the integration range and, in turn, for significantly tighter bounds on the approximation error. Namely, we propose the clipping of the score function whenever it exceeds a suitably chosen upper bound. In the vast majority of cases, the corresponding clipped estimates of Fisher estimation are identical to their non-clipped counterparts, meaning that the clipping has a negligible influence on the estimation accuracy. However, the knowledge that extreme values of the score would have been clipped, had they occurred, allows for much-improved performance guarantees.

It should be explicitly stated that this paper does not address the question of how best to estimate Fisher information. Although this question is highly interesting and relevant, it is far beyond the scope of this work. In addition, it is not the aim of the paper to compare the plug-in estimator to alternative estimators for the Fisher information or to claim that it provides superior result. A variety of well-motivated parametric and nonparametric Fisher information estimators have been proposed in the literature; see, for example, [9,10,11] and the references therein. However, comparing and contrasting these estimators in a fair manner is not straightforward and arguably constitutes a research question in its own right. Finally, the problem of obtaining estimator-independent bounds on the sample complexity of Fisher information falls under the umbrella of estimation of nonlinear functionals; see, for example, [12]. Most of the commonly used information measures, such as entropy, relative entropy, and mutual information, are nonlinear functionals, and their estimation has recently received considerable attention; the interested reader is referred to [13,14,15,16,17] and the references therein.

Despite its limited scope, we are convinced that the work presented in this paper is useful in a wider context. First, from a theoretical point of view, it strengthens some classic results in nonparametric estimation and, as explained above, provides bounds for the finite-sample regime, thus filling a gap in the literature. Second, from a practical perspective, the Fisher information typically provides useful bounds or limits on the estimation error (e.g., the well-known Cramér–Rao lower bound), but is not in itself the quantity of interest—an exception is the case of estimating a random signal in additive Gaussian noise, where the minimum mean square error (MMSE) and other relevant quantities can be expressed in term of the Fisher information. The problem of estimating Fisher information also arises in image processing, model selection, experimental design, and many more areas. Applications of our results include, for example, to provide the Cramér–Rao bound and, for the case of a random variable in additive Gaussian noise, to address the power allocation problem [18]. These connections will be discussed in more detail in Section 4. Most often, however, Fisher information plays the role of side information, and its estimation does not warrant investing large computational resources. This prevents the use of sophisticated estimators, which require solving non-trivial optimization problems. In contrast, kernel density estimates are relatively easy to compute and have been widely used in nonparametric statistics so that efficient implementations in software or even hardware [19] are readily available. Hence, for the foreseeable future, plug-in estimators are bound to remain a common and often the only viable option for estimating Fisher information in practice.

The paper is organized as follows: Section 2 revisits Bhattacharya’s estimator. In particular, Theorem 1 provides explicit and tighter non-asymptotic bounds on its convergence rate, improving the results in [1,2]. Furthermore, Theorem 2 provides an alternative bound under the additional assumption that the density function is upper bounded within any given interval. The explicit non-asymptotic results enable us to see that the sample complexity of Bhattacharya’s estimator is considerable and that the potentially unbounded score function is a critical bottleneck for tighter bounds. Section 3 proposes a “harmless” modification of Bhattacharya’s estimator, namely, a clipping of the estimated score function, which is shown to be sufficient to remedy its large sample complexity. In particular, Theorem 3 shows that the clipped estimator has significantly better bounds on rates of convergence, albeit with slightly different assumptions on the PDF. Section 4 evaluates the convergence rates of the two estimators for the practically relevant case of a random variable contaminated by additive Gaussian noise. Moreover, using Brown’s identity, which relates the Fisher information and the MMSE, consistent estimators for the MMSE are proposed and their rates of convergence are evaluated in Proposition 1. Section 5 concludes the paper.

### Notation

The expected value and variance of a random variable *X* are denoted by E[X] and Var(X), respectively. The gamma function is denoted by Γ(·). Estimators of a PDF *f* based on *n* samples are denoted by $f_{n}$. No notational distinction is made between an estimator, which is a random variable, and its realizations (estimates), which are deterministic. However, the difference will be clear from the context or will be highlighted explicitly otherwise. The *n*th derivative of a function F:R→R is denoted by $F^{\left(n\right)}$; the first-order derivative is also denoted by $F^{\prime }$ to improve readability.

## 2. Bhattacharya’s Estimator

In this section, we revisit the asymptotically consistent estimator proposed by Bhattacharya in [1] and produce explicit and non-asymptotic bounds on its accuracy.

Bhattacharya’s estimator is given by

(2) $$I_{n}=\int_{-k_{n}}^{k_{n}}\frac{{\left(f_{n}^{\prime }\left(t\right)\right)}^{2}}{f_{n}\left(t\right)}dt,$$

where $k_{n}\geq 0$ determines the integration interval as a function of the sample size *n* and the unknown functions *f* and $f^{\prime }$ are replaced by their kernel estimates, that is,

(3) $$\begin{array}{cc} f_{n}\left(t\right) & =\frac{1}{n}\sum_{i=1}^{n}\frac{1}{a_{0}}K\left(\frac{t-Y_{i}}{a_{0}}\right), \end{array}$$

(4) $$\begin{array}{cc} f_{n}^{\prime }\left(t\right) & =\frac{1}{n}\sum_{i=1}^{n}\frac{1}{a_{1}}K^{\prime }\left(\frac{t-Y_{i}}{a_{1}}\right). \end{array}$$

Here, $a_{0},a_{1}>0$ are bandwidth parameters, and K:R→R denotes the kernel, which is assumed to satisfy certain regularity conditions that will be discussed later in this section.

### 2.1. Estimating a Density and Its Derivatives

In order to analyze plug-in estimators, it is necessary to obtain rates of convergence for $f_{n}$ and $f_{n}^{\prime }$, that is, the kernel estimators of the density and its derivative. The following result, which is largely based on the proof by Schuster in [3], provides such rates. The proof in [3] makes use of the Dvoretzky–Kiefer–Wolfowitz (DKW) inequality for the empirical CDF. The next lemma refines the result in [3] by using the best possible constant for the DKW inequality shown in [20].

**Lemma** **1.** *Let r∈{0,1} and*

(5) $$\begin{array}{cc} v_{r} & =\int_{-\infty }^{\infty }\left|K^{\left(r+1\right)}\left(t\right)\right|dt, \end{array}$$

(6) $$\begin{array}{cc} \delta_{r,a_{r}} & =\underset{t\in R}{\sup}\left|E\left[f_{n}^{\left(r\right)}\left(t\right)\right]-f^{\left(r\right)}\left(t\right)\right|. \end{array}$$

*Then, for any $\epsilon >\delta_{r,a_{r}}$ and any n≥1, the following bound holds:*

(7) $$P\left[\underset{t\in R}{\sup}\left|f_{n}^{\left(r\right)}\left(t\right)-f^{\left(r\right)}\left(t\right)\right|>\epsilon \right]\leq 2e^{-2n\frac{a_{r}^{2r+2}{\left(\epsilon -\delta_{r,a_{r}}\right)}^{2}}{v_{r}^{2}}}.$$

**Proof.**  See Appendix A. □

### 2.2. Analysis of Bhattacharya’s Estimator

The following theorem is a non-asymptotic refinement of the result obtained by Bhattacharya in Theorem 3 of [1] and Dmitriev and Tarasenko in Theorem 1 of [2].

**Theorem** **1.** *Assume that there exists a function ϕ:R→R such that*

(8) $$\underset{|t|\leq x}{\sup}\frac{1}{f(t)}\leq \phi \left(x\right),\;\forall x\in R.$$

*Then, provided that*

(9) $$\underset{\left|t\right|\leq k_{n}}{\sup}\left|f_{n}^{\left(r\right)}\left(t\right)-f^{\left(r\right)}\left(t\right)\right|\leq \epsilon_{r},\;r\in \left\{0,1\right\},$$

*and*

(10) $$\epsilon_{0}\phi \left(k_{n}\right)<1,$$

*the following bound holds:*

(11) $$\left|I\left(f\right)-I_{n}\right|\;\leq \frac{4\epsilon_{1}k_{n}\rho_{\max}\left(k_{n}\right)+2\epsilon_{1}^{2}k_{n}\phi \left(k_{n}\right)+\epsilon_{0}\phi \left(k_{n}\right)I\left(f\right)}{1-\epsilon_{0}\phi \left(k_{n}\right)}+c\left(k_{n}\right),$$

*where*

(12) $$\begin{array}{cc} \rho_{\max}\left(k_{n}\right) & =\underset{\left|t\right|\leq k_{n}}{\sup}\left|\frac{f^{\prime }\left(t\right)}{f(t)}\right|, \end{array}$$

(13) $$\begin{array}{cc} c(k_{n}) & =I\left(f\right)-\int_{-k_{n}}^{k_{n}}\frac{{\left(f^{\prime }\left(t\right)\right)}^{2}}{f(t)}dt. \end{array}$$

**Proof.**  See Appendix B. □

The bound in (11) is an improvement of the original bound in [1,2], which contains terms of the form $\epsilon_{0}\phi^{4}\left(k_{n}\right)$.

Note that $\phi (k_{n})$ in (8) can be rapidly increasing with $k_{n}$. For example, as will be shown later, $\phi (k_{n})$ increases super-exponentially with $k_{n}$ for a random variable contaminated by Gaussian noise. This implies that, while Bhattacharya’s estimator converges, the rate of convergence guaranteed by the bound in (11) is extremely slow. A modified bound is proposed in the subsequent theorem.

**Theorem** **2.** *Assume that f(t) is bounded on the interval $t\in [-k_{n},k_{n}]$, i.e.,*

(14) $$\underset{\left|t\right|\leq k_{n}}{\sup}f\left(t\right)\leq f_{0},$$

*for some $f_{0}\in R$. If the assumptions in (8), (9), and (10) hold, then*

(15) $$|I\left(f\right)-I_{n}|\leq \left[\epsilon_{1}\left(4+d_{f}\left(k_{n}\right)+d_{f_{n}}\left(k_{n}\right)\right)+\epsilon_{0}\left(2+d_{f_{n}}\left(k_{n}\right)\right)\rho_{\max}\left(k_{n}\right)\right]\psi \left(\epsilon_{0},k_{n}\right)+c\left(k_{n}\right),$$

*where $\rho_{\max}$ and c are given by (12) and (13), respectively,*

(16) $$\psi \left(\epsilon_{0},k_{n}\right)=\max\left(\log\left(f_{0}+\epsilon_{0}\right),\log\left(\frac{\phi (k_{n})}{1-\epsilon_{0}\phi \left(k_{n}\right)}\right)\right),$$

*and $d_{g}\left(k_{n}\right)$ denotes the number of zeros of the derivative of the function g on the interval $\left[-k_{n},k_{n}\right]$, i.e.,*

(17) $$d_{g}\left(k_{n}\right)=\left|\left\{t\in \left[-k_{n},k_{n}\right]:g^{\prime }\left(t\right)=0\right\}\right|.$$

**Proof.**  See Appendix C. □

**Remark** **1.** *Note that ψ in (15) is on the order of $\log(\phi \left(k_{n}\right))$, which typically increases much more slowly with $k_{n}$ than ϕ in (11). As a result, the bound in Theorem 2 can lead to a better bound on the convergence rate than that in Theorem 1, given appropriate upper bounds on $d_{f}$ and $d_{f_{n}}$. Since Gaussian blurring of a univariate density function never creates new maxima, we have that $d_{f_{Y}}\leq d_{f_{X}}$, which is a constant. However, to the best of our knowledge, the only known upper bound on $d_{f_{n}}$ is given by $d_{f_{n}}\leq n$ [21] (Theorem 2), which is not useful in practice. Despite this drawback, we include Theorem 2 for the sake of completeness and in the hope that tighter bounds on $d_{f_{n}}$ might be established in the future.*

The main problem in the convergence analysis of the estimator in (2) is that $1/f_{n}\left(t\right)$ is only bounded if $f\left(t\right)>\epsilon_{0}$. For distributions with sub-Gaussian tails, this implies that the interval $\left[-k_{n},k_{n}\right]$, on which this is guaranteed to be the case, grows sub-logarithmically (compare Theorem 4), causing the required number of samples to grow super-exponentially. In next section, we propose an estimator that has better guaranteed rates of convergence.

## 3. The Clipped Bhattacharya Estimator

In order to remedy the slow guaranteed convergence rates of Bhattacharya’s estimator, we dispense with the tail assumption in (8), but introduce the new assumption that the unknown true score function $\rho \left(t\right)=f^{\prime }\left(t\right)/f\left(t\right)$ is bounded (in absolute value) by a known function $\bar{\rho }$. This allows us to clip $f_{n}^{\prime }\left(t\right)/f_{n}\left(t\right)$ and, in turn, $1/f_{n}\left(t\right)$ without affecting the consistency of the estimator.

**Theorem** **3.** *Assume that there exists a function $\bar{\rho }:R\to R$ such that*

(18) $$\left|\rho \left(t\right)\right|\leq \left|\bar{\rho }\left(t\right)\right|,\;\forall t\in R$$

*and let*

(19) $$I_{n}^{c}=\int_{-k_{n}}^{k_{n}}\min\left\{\left|\rho_{n}\left(t\right)\right|,\;\left|\bar{\rho }\left(t\right)\right|\right\}\;\left|f_{n}^{\prime }\left(t\right)\right|\;dt,$$

*where*

(20) $$\rho_{n}\left(t\right)=\frac{f_{n}^{\prime }\left(t\right)}{f_{n}\left(t\right)}.$$

*Under the assumptions in (9), it holds that*

(21)|I(f)−Inc|≤max4ϵ1Φ1(kn)+2ϵ0Φ2(kn)+c(kn),3ϵ1Φmax1(kn)+ϵ0Φmax2(kn)(22)≤4ϵ1Φmax1(kn)+2ϵ0Φmax2(kn)+c(kn),

*where $c(k_{n})$ is defined in (13) and*

(23) $$\begin{array}{cc} \Phi^{m}\left(x\right) & =\int_{-x}^{x}\left|\rho^{m}\left(t\right)\right|\;dt, \end{array}$$

(24) $$\begin{array}{cc} \Phi_{\max}^{m}\left(x\right) & =\int_{-x}^{x}\left|{\bar{\rho }}^{m}\left(t\right)\right|\;dt. \end{array}$$

*In addition, if f(t) is bounded as in (14), then*

(25) $$\Phi^{m}\left(k_{n}\right)\leq \min\left\{\left(2+d_{f}\right){\bar{\rho }}^{m-1}\left(k_{n}\right)\psi \left(0,k_{n}\right)\;,\;\Phi_{\max}^{m}\left(k_{n}\right)\right\},$$

*where ψ and $d_{f}$ are defined in (16) and (17), respectively.*

**Proof.**  See Appendix D. □

For the upper-bound function $\bar{\rho }\left(t\right)$ in assumption (18), in practice, we can set $\bar{\rho }\left(k_{n}\right)=\rho_{\max}\left(k_{n}\right)$ if the latter is available. Although $\rho_{\max}\left(k_{n}\right)$ also increases with $k_{n}$, it usually increases much more slowly than $\phi (k_{n})$. For example, as shown later, $\rho_{\max}\left(k_{n}\right)$ is linear in $k_{n}$ in the Gaussian noise case. As a result, better bounds on the convergence rate can be shown for the clipped estimator.

## 4. Estimation of the Fisher Information of a Random Variable in Gaussian Noise

This section evaluates the results of Section 2 and Section 3 for the important special case of a random variable contaminated by additive Gaussian noise. To this end, we let $f_{Y}$ denote the PDF of a random variable

(26) $$Y=\sqrt{\mathrm{snr}}X+Z,$$

where snr>0 is a signal-to-noise-ratio parameter, *X* is an arbitrary random variable, *Z* is a standard Gaussian random variable, and *X* and *Z* are independent. We are interested in estimating the Fisher information of $f_{Y}$. We only make the very mild assumption that *X* has a finite second moment, but otherwise, it is allowed to be an arbitrary random variable. We further assume that snr is known and that Gaussian kernels are used in the density estimators, i.e.,

(27) $$K\left(t\right)=\frac{1}{\sqrt{2\pi }}e^{-\frac{t^{2}}{2}}.$$

The following lemma provides explicit expressions for the quantities appearing in Section 2 and Section 3 that are needed to evaluate the error bounds for the Bhattacharya and the clipped estimator.

**Lemma** **2.** *Let K be as in (27). Then,*

(28) $$\begin{array}{cc} \delta_{r,a_{r}} & \leq a_{r}\cdot \left\{\begin{array}{cc} \frac{1}{\pi \sqrt{e}}, & r=0\\ \frac{\frac{2}{e}+1}{\pi }, & r=1, \end{array}\right. \end{array}$$

(29) $$\begin{array}{cc} v_{r} & =\left\{\begin{array}{cc} \sqrt{\frac{2}{\pi }}, & r=0\\ \sqrt{\frac{2}{e\pi }}, & r=1, \end{array}\right. \end{array}$$

(30) $$\begin{array}{cc} \rho_{\max}\left(k_{n}\right) & \leq \sqrt{3\mathrm{snr}\mathrm{Var}(X)}+3k_{n}, \end{array}$$

(31) $$\begin{array}{cc} I(f_{Y}) & \leq 1, \end{array}$$

(32) $$\begin{array}{cc} \phi (t) & \leq \sqrt{2\pi }e^{t^{2}+\mathrm{snr}\;E[X^{2}]}. \end{array}$$

**Proof.**  See Appendix F. □

We now bound $c(k_{n})$. To this end, we need the notion of sub-Gaussian random variables: A random variable *X* is said to be α-sub-Gaussian if

(33) $$E\left[e^{tX}\right]\leq e^{\frac{\alpha^{2}t^{2}}{2}}\;\forall t\in R.$$

**Lemma** **3.** *Suppose that $E[X^{2}]<\infty$. Then,*

(34) $$c\left(k_{n}\right)\leq \underset{v>0}{\inf}\frac{2\Gamma^{\frac{1}{\left(1+v\right)}}\left(v+\frac{1}{2}\right)}{\pi^{\frac{1}{2(1+v)}}}{\left(\frac{\mathrm{snr}\;E[X^{2}]+1}{k_{n}^{2}}\right)}^{\frac{v}{1+v}}.$$

*In addition, if |X| is α-sub-Gaussian, then*

(35) $$c\left(k_{n}\right)\leq \underset{v>0}{\inf}\frac{2\Gamma^{\frac{1}{\left(1+v\right)}}\left(v+\frac{1}{2}\right)}{\pi^{\frac{1}{2(1+v)}}}{\left(2e^{\frac{\alpha^{2}\mathrm{snr}-k_{n}^{2}}{2}}\right)}^{\frac{v}{1+v}}.$$

**Proof.**  See Appendix G. □

### 4.1. Convergence of Bhattacharya’s Estimator

By combining the results in Lemma 1, Theorem 1, Lemma 2, and Lemma 3, we have the following theorem.

**Theorem** **4.** *Let K be as in (27). Choose the parameters of Bhattacharya’s estimator as follows: $a_{0}=n^{-w_{0}}$, where $w_{0}\in \left(0,\frac{1}{4}\right)$, $a_{1}=n^{-w_{1}}$, where $w_{1}\in \left(0,\frac{1}{6}\right)$, and $k_{n}=\sqrt{u\log(n)}$, where $u\in \left(0,\min(w_{0},w_{1})\right)$. Then, for $n^{w_{0}-u}>c_{5}$,*

(36) $$P\left[\left|I_{n}-I\left(f_{Y}\right)\right|\geq \varepsilon_{n}\right]\leq 2e^{-c_{1}n^{1-4w_{0}}}+2e^{-c_{2}n^{1-6w_{1}}},$$

*where*

(37) $$\varepsilon_{n}\leq \frac{n^{-w_{1}}\sqrt{u\log(n)}\left(4c_{3}+12\sqrt{u\log(n)}+2c_{5}n^{u-w_{1}}\right)+c_{5}n^{u-w_{0}}}{1-c_{5}n^{u-w_{0}}}+\frac{c_{4}}{\sqrt{u\log(n)}},$$

*and where the constants are given by*

(38) $$\begin{array}{cc} c_{1} & =\pi {\left(1-\frac{1}{\sqrt{2\pi e}}\right)}^{2}, \end{array}$$

(39) $$\begin{array}{cc} c_{2} & =e\pi {\left(1-\frac{\frac{2}{e}+1}{\sqrt{2\pi }}\right)}^{2}, \end{array}$$

(40) $$\begin{array}{cc} c_{3} & =\sqrt{3\mathrm{snr}\mathrm{Var}(X)}, \end{array}$$

(41) $$\begin{array}{cc} c_{4} & =\frac{2\Gamma^{\frac{1}{2}}\left(\frac{3}{2}\right)\sqrt{\mathrm{snr}\;E[X^{2}]+1}}{\pi^{\frac{1}{4}}}, \end{array}$$

(42) $$\begin{array}{cc} c_{5} & =\sqrt{2\pi }e^{\mathrm{snr}\;E\left[X^{2}\right]}. \end{array}$$

*In addition, if |X| is α-sub-Gaussian, then*

(43) $$\varepsilon_{n}\leq \frac{n^{-w_{1}}\sqrt{u\log(n)}\left(4c_{3}+12\sqrt{u\log(n)}+2c_{5}n^{u-w_{1}}\right)+c_{5}n^{u-w_{0}}}{1-c_{5}n^{u-w_{0}}}+c_{6}n^{-\frac{u}{4}},$$

*where*

(44) $$c_{6}=\frac{2^{\frac{3}{2}}\Gamma^{\frac{1}{2}}\left(\frac{3}{2}\right)e^{\frac{\alpha^{2}\mathrm{snr}}{4}}}{\pi^{\frac{1}{4}}}.$$

**Proof.**  See Appendix H. □

Note that the parameters $k_{n}$, $a_{0}$, and $a_{1}$ are chosen so as to guarantee the convergence of $I_{n}\left(f_{n}\right)$ to $I(f_{Y})$ with probability 1. For the details, please refer to the proof in Appendix H.

The parameters *u* and *w* in the above theorem are auxiliary variables that couple the bandwidth of the kernel density estimators in (3) and (4) with the integration range of the Fisher information estimator in (2). Choosing them according to Theorem 4 results in a trade-off between precision, $\varepsilon_{n}$, and confidence, i.e., the probability of the estimation error exceeding $\varepsilon_{n}$. On the one hand, small values of *u* and large values of *w* result in better precision (i.e., small $\varepsilon_{n}$) at the cost of a lower confidence (i.e., large probability of exceeding $\varepsilon_{n}$). On the other hand, large values of *u* and small values of *w* improve the confidence but deteriorate the precision. In turn, this also affects the convergence rates, meaning that faster convergence of the precision can be achieved at the expense of a slower convergence of the confidence and vice versa.

### 4.2. Convergence of the Clipped Estimator

From the evaluation of Bhattacharya’s estimator in Theorem 4, it is apparent that the bottleneck term is the truncation parameter $k_{n}=\sqrt{u\log(n)}$, which results in slow precision decay of the order $\varepsilon_{n}=O\left(\frac{1}{\sqrt{u\log(n)}}\right)$. Next, it is shown that the clipped estimator results in improved precision over Bhattacharya’s estimator. Specifically, the precision will be shown to decay polynomially in *n* instead of logarithmically. Another benefit of the clipped estimator is that its error analysis holds for every n≥1.

By utilizing the results in Lemma 1, Lemma 2, and Lemma 3, we specialize the result in Theorem 2 to the Gaussian noise case.

**Theorem** **5.** *Let K be as in (27). Choose the parameters of the clipped estimator as follows: $a_{0}=n^{-w_{0}}$, where $w_{0}\in \left(0,\frac{1}{4}\right)$, $a_{1}=n^{-w_{1}}$, where $w_{1}\in \left(0,\frac{1}{6}\right)$, and $k_{n}=n^{u}$, where $u\in \left(0,\min\left(\frac{w_{0}}{3},\frac{w_{1}}{2}\right)\right)$. Then, for n≥1*

(45) $$P\left[\left|I_{n}^{c}-I\left(f_{Y}\right)\right|\geq \varepsilon_{n}\right]\leq 2e^{-c_{1}n^{1-4w_{0}}}+2e^{-c_{2}n^{1-6w_{1}}},$$

*where*

(46) $$\varepsilon_{n}\leq 4n^{3u-w_{0}}\left(c_{3}^{2}n^{-2u}+3\right)+4n^{2u-w_{1}}\left(2c_{3}n^{-u}+3\right)+c_{4}n^{-u},$$

*and the constants $c_{1}$ to $c_{4}$ are as in Theorem 4. In addition, if |X| is α-sub-Gaussian, then*

(47) $$\varepsilon_{n}\leq 4n^{3u-w_{0}}\left(c_{3}^{2}n^{-2u}+3\right)+4n^{2u-w_{1}}\left(2c_{3}n^{-u}+3\right)+c_{6}e^{-\frac{n^{2u}}{4}},$$

*where $c_{6}$ is given by (44).*

**Proof.**  See Appendix I. □

Again, the parameters $k_{n}$, $a_{0}$, and $a_{1}$ are chosen to guarantee the consistency of the estimator. For further details, please refer to Appendix I.

### 4.3. Applications to the Estimations of the MMSE

As discussed in the introduction, the Fisher information is often merely a proxy for the actual quantity of interest. One accuracy measure that is typically of interest is the MMSE, which is defined as

(48) $$\mathrm{mmse}\left(X|Y\right)=E\left[{\left(X-E\left[X|Y\right]\right)}^{2}\right].$$

In additive Gaussian noise, the MMSE can not only be bounded by the Fisher information, but both are related via Brown’s identity:

(49) $$I(f_{Y})=1-\mathrm{snr}\;\mathrm{mmse}(X|Y).$$

Based on this relation, we propose the following estimators for the MMSE:

(50) $${\mathrm{mmse}}_{n}\left(X,\mathrm{snr}\right)=\frac{1-I_{n}}{\mathrm{snr}}$$

and

(51) $${\mathrm{mmse}}_{n}^{c}\left(X,\mathrm{snr}\right)=\frac{1-I_{n}^{c}}{\mathrm{snr}}.$$

The results for the estimators of Fisher information in Theorem 4 and Theorem 5 can be immediately extended to the MMSE estimators as follows.

**Proposition** **1.** *Let K be as in (27), and let $w_{0},w_{1}$, and n be such that they satisfy the conditions in Theorem 4. It then holds that*

(52) $$P\left[\left|{\mathrm{mmse}}_{n}\left(X,\mathrm{snr}\right)-\mathrm{mmse}\left(X,\mathrm{snr}\right)\right|\geq \mathrm{snr}\varepsilon_{n}\right]\leq 2e^{-c_{1}n^{1-4w_{0}}}+2e^{-c_{2}n^{1-6w_{1}}},$$

*where $\varepsilon_{n}$, $c_{1}$, and $c_{2}$ are given in Theorem 4.*

**Proposition** **2.** *Let K be as in (27), and let $w_{0}$ and $w_{1}$ be such that they satisfy the conditions in Theorem 5. It then holds that*

(53) $$P\left[\left|{\mathrm{mmse}}_{n}^{c}\left(X|Y\right)-\mathrm{mmse}\left(X|Y\right)\right|\geq \mathrm{snr}\varepsilon_{n}\right]\leq 2e^{-c_{1}n^{1-4w_{0}}}+2e^{-c_{2}n^{1-6w_{1}}},$$

*where $\varepsilon_{n}$, $c_{1}$, and $c_{2}$ are given in Theorem 5.*

### 4.4. Sample Complexity

Finally, we demonstrate the difference in the bounds on the convergence rates between Bhattacharya’s estimator and its clipped version by comparing the sample complexity of the two estimators, that is, the required number of samples to guarantee a given accuracy with a given confidence. MATLAB implementations of both estimators, as well as the code used to generate the figures below, can be found in [22].

To this end, we consider the simple example of estimating the density of a Gaussian random variable in additive Gaussian noise. More precisely, we assume that *X* and *Z* in (26) are independent and identically distributed according to the standard normal distribution N(0,1), and that snr=1. This trivially implies that *X* is α-sub-Gaussian with α=1. In order to make the comparison as fair as possible, the parameters of the kernel estimators, $a_{0}$, $a_{1}$, and $k_{n}$, are not chosen according to Theorem 4 or Theorem 5, but are calculated by numerically minimizing the required number of samples; see [22] for details.

**Figure 1:**
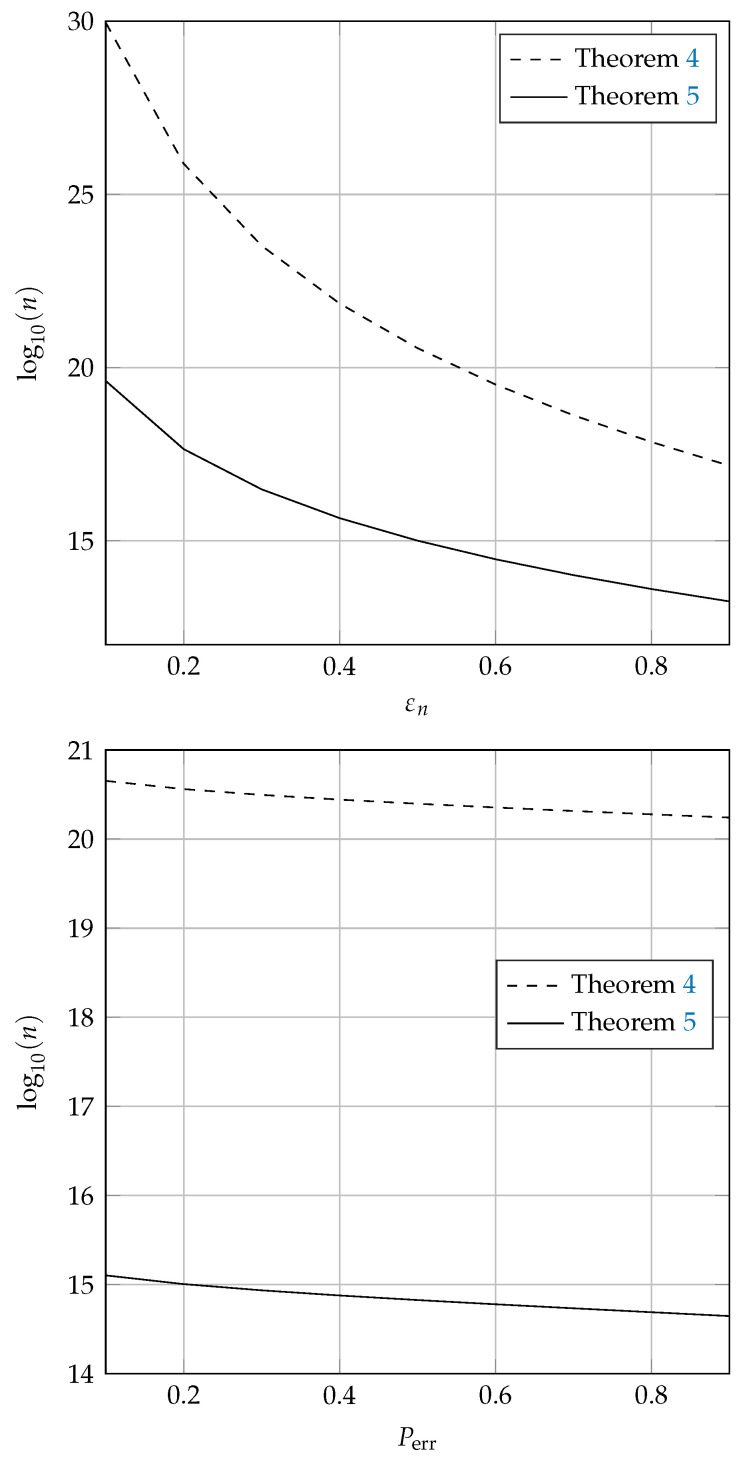
Sample complexity with Gaussian input. Left: number of samples required versus error of the estimators $I_{n}$ and $I_{n}^{c}$ given $P_{\mathrm{err}}=0.2$. Right: number of samples required versus confidence of the estimators with given $\varepsilon_{n}=0.5$.

Let $P_{\mathrm{err}}=P\left[\left|I_{n}-I\left(f_{Y}\right)\right|\geq \varepsilon_{n}\right]$. The left-hand plot in Figure 1 shows the corresponding bounds on the sample complexities of the two estimators with $P_{\mathrm{err}}=0.2$ and $\varepsilon_{n}$ varying from 0.1 to 0.9. Note that the results with larger $\varepsilon_{n}$ are not shown because $I(f_{Y})\leq 1$, as shown in Lemma 2. Moreover, the right-hand plot in Figure 1 shows the sample complexities for $\varepsilon_{n}=0.5$ with $P_{\mathrm{err}}$ varying from 0.1 to 0.9. By inspection, the clipped estimator reduces the sample complexity by several orders of magnitude; note that the *y*-axes scale logarithmically. As discussed before, this does not imply that the clipped estimator is more accurate in general. However, it does imply that the clipped estimator provides significantly better worst-case performance, i.e., it requires significantly fewer samples to guarantee a certain precision or confidence. Finally, note that this improvement comes at a low cost in terms of complexity and regularity assumptions. The complexity of both algorithms is almost identical, with the clipped estimator only requiring an additional evaluation of $\bar{\rho }$. The regularity conditions are identical for bounded density functions, and slightly stronger for the clipped estimator for unbounded density functions.

## 5. Conclusions

This work focused on the estimation of the Fisher information for the location of a univariate random variable using plug-in estimators based on estimators of the PDF and its derivative. Two estimators of the Fisher information were considered. The first estimator is the estimator due to Bhattacharya, for which new, sharper convergence results were shown. The paper also proposed a second estimator, termed a clipped estimator, which provides better bounds on the convergence rates. The accuracy bounds on both estimators were specialized to the practically relevant case of a random variable contaminated by additive Gaussian noise. Moreover, using special proprieties of the Gaussian noise case, two estimators for the MMSE were proposed, and their convergence rates were analyzed. This was done by using Brown’s identity, which connects the Fisher information and the MMSE. Finally, using a numerical example, it was demonstrated that the proposed clipped estimator can achieve a significantly lower sample complexity at little or no additional cost.

## Data Availability

Data sharing not applicable.

## Appendix A. A Proof of Lemma 1

Our starting point is the following bound due to [3] (p. 1188):

(A1) $$\underset{t\in R}{\sup}\left|E\left[f_{n}^{\left(r\right)}\left(t\right)\right]-f_{n}^{\left(r\right)}\left(t\right)\right|\leq \frac{v_{r}}{a_{r}^{r+1}}\underset{t\in R}{\sup}\left|F_{n}\left(t\right)-F_{Y}\left(t\right)\right|,$$

where *F* is the CDF of *f*, $F_{n}$ is the empirical CDF, and $v_{r}$ is defined in (5). Now, let $\delta_{r,a_{r}}$ be as in (6), and consider the following sequence of bounds:

(A2)Psupt∈Rfn(r)(t)−f(r)(t)>ϵ≤Psupt∈Rfn(r)(t)−E[fn(r)(t)]>ϵ−δr,ar(A3)≤Psupt∈R|Fn(t)−F(t)|>arr+1(ϵ−δr,ar)vr(A4)≤2e−2nar2r+2(ϵ−δr,ar)2vr2,

where (A2) follows by using the triangle inequality; (A3) follows by using the bound in (A1); and (A4) follows by using the sharp DKW inequality [20]:

(A5) $$P\left[\underset{t\in R}{\sup}\left|F_{n}\left(t\right)-F\left(t\right)\right|>\epsilon \right]\leq 2e^{-2n\epsilon^{2}}.$$

This concludes the proof.

## Appendix B. A Proof of Theorem 1

First, using the triangle inequality, we have that

(A6) $$\left|I\left(f\right)-I_{n}\right|\;\leq \;\left|\int_{-k_{n}}^{k_{n}}\frac{{\left(f_{n}^{\prime }\left(t\right)\right)}^{2}}{f_{n}\left(t\right)}-\frac{{\left(f^{\prime }\left(t\right)\right)}^{2}}{f(t)}dt\right|+c\left(k_{n}\right).$$

Next, we bound the first term in (A6):

 ∫−knkn(fn′(t))2fn(t)−(f′(t))2f(t)dt(A7)= ∫−knknf(t)(fn′(t))2−fn(t)(f′(t))2fn(t)f(t)dt(A8)≤ ∫−knknf(t)(fn′(t))2−f(t)(f′(t))2fn(t)f(t)dt + ∫−knknf(t)(f′(t))2−fn(t)(f′(t))2fn(t)f(t)dt(A9)= ∫−knkn(fn′(t))2−(f′(t))2fn(t)dt + ∫−knknfn(t)−f(t)fn(t)(f′(t))2f(t)dt(A10)≤sup|t|≤kn|fn′(t)+f′(t)|fn(t)fn(t)−f(t)2kn+sup|t|≤kn|fn(t)−f(t)|fn(t)∫−knkn(f′(t))2f(t)dt(A11)≤sup|t|≤kn|fn′(t)+f′(t)|fn(t)ϵ12kn+sup|t|≤kn1fn(t)ϵ0I(f),

where the last bound follows from the assumptions in (9). Now, consider the first term in (A11):

(A12)sup|t|≤kn|fn′(t)+f′(t)|fn(t)≤sup|t|≤kn2|f′(t)|+ϵ1fn(t)(A13)≤sup|t|≤kn2|f′(t)|+ϵ1f(t)−f(t)+fn(t)(A14)≤sup|t|≤kn2|f′(t)|+ϵ1f(t)−ϵ0(A15)=sup|t|≤kn2f′(t)f(t)+ϵ1f(t)1−ϵ0f(t)(A16)≤2sup|t|≤knf′(t)f(t)+ϵ1ϕ(kn)1−ϵ0ϕ(kn),

where the bound in (A14) follows from the assumptions in (9) and the properties of ϕ, which imply

(A17) $$\epsilon_{0}\phi \left(k_{n}\right)<1\;\Rightarrow \;\epsilon_{0}<f\left(t\right),\;\forall \left|t\right|\leq k_{n};$$

and the bound in (A16) follows from the definition of ϕ in (8). Now, consider the second term in (A11):

(A18)sup|t|≤kn1fn(t)=sup|t|≤kn1f(t)−f(t)+fn(t)(A19)≤sup|t|≤kn1f(t)−ϵ0(A20)=sup|t|≤kn11−ϵ0f(t)1f(t)(A21)≤11−ϵ0ϕ(kn)ϕ(kn),

where (A20) follows by using similar steps, leading to the bound in (A14), and (A21) follows from the definition of ϕ.

Combining the bounds in (A6), (A11), (A16), and (A21) concludes the proof.

## Appendix C. A Proof of Theorem 2

Using (9), (14), and (A21), it holds that

(A22)|log(fn)|≤maxlog(fn),log1fn(A23)≤maxlog(f0+ϵ0),logϕ(kn)1−ϵ0ϕ(kn)(A24)=ψ(ϵ0,kn).

Next, we bound the first term on the right-hand side of (A6). Starting with (A9) above, it holds that

 ∫−knkn(fn′(t))2fn(t)−(f′(t))2f(t)dt(A25)≤ϵ1∫−knknfn′(t)+f′(t)fn(t)dt+ϵ0∫−knkn(f′(t))2fn(t)f(t)dt(A26)≤ϵ1∫−knknfn′(t)fn(t)dt+ϵ1∫−knknf′(t)fn(t)dt+ϵ0ρmax(kn)∫−knknf′(t)fn(t)dt,

where the inequality in (A25) follows again from (9), and the last bound follows from the triangle inequality together with the definition of $\rho_{\max}$.

Consider the integral in the first term in (A26):

 ∫−knknfn′(t)fn(t)dt(A27)=∫−knkn∇log(fn(t))dt(A28)=∫−knknsign∇log(fn(t))·∇log(fn(t))dt(A29)=sign∇log(fn(t))·log(fn(t))|−knkn−∫−knknlog(fn(t))ddtsign∇log(fn(t))dt,

where the last equality follows from integration by parts. The first term in (A29) can be upper bounded as

(A30) $$\mathrm{sign}\left(\nabla \log(f_{n}\left(t\right))\right)\cdot \log\left(f_{n}\left(t\right)\right){|}_{-k_{n}}^{k_{n}}\;\leq 2\psi \left(\epsilon_{0},k_{n}\right),$$

where the inequality in (A30) follows from (A24). The second term in (A29) is given by

(A31)−∫−knknlog(fn(t))ddtsign∇log(fn(t))dt=−∑t∈[−kn,kn]:fn′(t)=0log(fn(t))(A32)≤dfn(kn)ψ(ϵ0,kn).

By substituting (A30) and (A32) into (A29), one obtains

(A33) $$\begin{array}{c} \int_{-k_{n}}^{k_{n}}\left|\frac{f_{n}^{\prime }\left(t\right)}{f_{n}\left(t\right)}\right|dt\leq \left(2+d_{f_{n}}\right)\psi \left(\epsilon_{0},k_{n}\right). \end{array}$$

Next, we consider the integral in the second and third terms in (A26):

(A34)∫−knknf′(t)fn(t)dt≤∫−knknf′(t)f(t)−ϵ0dt(A35)=∫−knkn∇log(f(t)−ϵ0)dt(A36)=∫−knknsign∇log(f(t)−ϵ0)·∇log(f(t)−ϵ0)dt =sign∇log(f(t)−ϵ0)·log(f(t)−ϵ0)|−knkn(A37)−∫−knknlog(f(t)−ϵ0)ddtsign∇log(f(t)−ϵ0)dt(A38)≤(2+df(kn))maxlog(f0−ϵ0),logϕ(kn)1−ϵ0ϕ(kn)(A39)≤(2+df(kn))ψ(ϵ0,kn),

where the inequalities in (A34) follows from the assumptions in (9) and (A17), and the bound in (A38) follows by using steps similar to those leading to the bound in (A33).

Combining the bounds in (A6), (A26), (A33), and (A38) concludes the proof.

## Appendix D. A Proof of Theorem 3

The difficulty in bounding the error of a clipped estimator is in showing that the clipping is strict enough to avoid gross overestimation, yet permissive enough to avoid gross underestimation. The proof presented here is based on two auxiliary estimators that are constructed to under- and overestimate $I_{n}^{c}\left(f_{n}\right)$ in a controlled manner.

Let

(A40) $${\underline{I}}_{n}=\int_{-k_{n}}^{k_{n}}\frac{{\left\lceil f_{n}^{\prime }\left(t\right)-\epsilon_{1}\right\rfloor }^{2}}{f_{n}\left(t\right)+\epsilon_{0}}\;dt,$$

where ⌈•−ϵ⌋ denotes an “ϵ-compression” operator, i.e.,

(A41) $$\begin{array}{c} \left\lceil f\left(t\right)-\epsilon \right\rfloor =\;\left\{\begin{array}{cc} f(t)-\epsilon , & f(t)>\epsilon \\ 0, & -\epsilon \leq f(t)\leq \epsilon \\ f(t)+\epsilon , & f(t)<-\epsilon . \end{array}\right. \end{array}$$

Next, consider the estimator

(A42) $${\bar{I}}_{n}=\int_{-k_{n}}^{k_{n}}\frac{{\left\lceil f_{n}^{\prime }\left(t\right)-\gamma_{1,n}\left(t\right)\right\rfloor }^{2}}{f_{n}\left(t\right)+\gamma_{0,n}\left(t\right)}\;dt,$$

where the functions $\gamma_{i,n}:R\to \left[0,\epsilon_{i}\right]$, i=0,1 are chosen as follows: If it holds that

(A43) $$\left|\rho_{n}\left(t\right)\right|\leq \left|\bar{\rho }\left(t\right)\right|,$$

then $\gamma_{0,n}\left(t\right)=\gamma_{1,n}\left(t\right)=0$. If, on the other hand,

(A44) $$\left|\rho_{n}\left(t\right)\right|>\left|\bar{\rho }\left(t\right)\right|,$$

then $\gamma_{0,n}\left(t\right)$ and $\gamma_{1,n}\left(t\right)$ are chosen such that

(A45) $$\frac{\left\lceil f_{n}^{\prime }\left(t\right)-\gamma_{1,n}\left(t\right)\right\rfloor }{f_{n}\left(t\right)+\gamma_{0,n}\left(t\right)}=\bar{\rho }\left(t\right).$$

Note that because

(A46) $$\left|\frac{\left\lceil f_{n}^{\prime }\left(t\right)-\epsilon_{1}\right\rfloor }{f_{n}\left(t\right)+\epsilon_{0}}\right|\leq \left|\rho \left(t\right)\right|\leq \left|\bar{\rho }\left(t\right)\right|,$$

this is always possible.

In Appendix E, it is shown that the following relations hold between the estimators defined above:

(A47) $$\begin{array}{cc} {\underline{I}}_{n} & \leq I(f), \end{array}$$

(A48) $$\begin{array}{cc} {\underline{I}}_{n} & \leq I_{n}^{c}, \end{array}$$

(A49) $$\begin{array}{cc} I_{n}^{c} & \leq {\bar{I}}_{n}+\epsilon_{1}\Phi_{\max}^{1}\left(k_{n}\right), \end{array}$$

(A50) $$\begin{array}{cc} I\left(f\right)-{\underline{I}}_{n} & \leq 4\epsilon_{1}\Phi^{1}\left(k_{n}\right)+2\epsilon_{0}\Phi^{2}\left(k_{n}\right)+c\left(k_{n}\right), \end{array}$$

(A51) $$\begin{array}{cc} {\bar{I}}_{n}-{\underline{I}}_{n} & \leq 2\epsilon_{1}\Phi_{\max}^{1}\left(k_{n}\right)+\epsilon_{0}\Phi_{\max}^{2}\left(k_{n}\right). \end{array}$$

The bound in Theorem 3 can now be obtained by bounding the under- and overestimation errors separately. For $I_{n}^{c}\leq I\left(f\right)$, it holds that

(A52)I(f)−Inc≤I(f)−I_n(A53)≤4ϵ1Φ1(kn)+2ϵ0Φ2(kn)+c(kn).

For $I_{n}^{c}>I\left(f\right)$, it hold that

(A54)Inc−I(f)≤I¯n−I_n+ϵ1Φmax1(kn)(A55)≤3ϵ1Φmax1(kn)+ϵ0Φmax2(kn).

The bound in (22) follows. Furthermore, following the same steps as those leading to the bound in (A33), the bound in (25) follows.

## Appendix E. A Proof of the Estimator Relations in Theorem 3

The bound in (A47) follows directly from the fact that under the assumptions in (9)

(A56) $$\frac{{\left\lceil f_{n}^{\prime }\left(t\right)-\epsilon_{1}\right\rfloor }^{2}}{f_{n}\left(t\right)+\epsilon_{0}}\leq \frac{{\left(f^{\prime }\left(t\right)\right)}^{2}}{f(t)}.$$

Analogously, (A48) follows from

(A57) $$\frac{{\left\lceil f_{n}^{\prime }\left(t\right)-\epsilon_{1}\right\rfloor }^{2}}{f_{n}\left(t\right)+\epsilon_{0}}\leq \left|\rho \left(t\right)\right|\left|\left\lceil f_{n}^{\prime }\left(t\right)-\epsilon_{1}\right\rfloor \right|\leq \left|\rho \left(t\right)\right|\left|f_{n}^{\prime }\left(t\right)\right|.$$

In order to show (A50), note that under the assumptions in (9), it holds that

(A58) $$\begin{array}{cc} \left|\left\lceil f_{n}^{\prime }\left(t\right)-\epsilon_{1}\right\rfloor \right| & \leq |f^{\prime }\left(t\right)|, \end{array}$$

(A59) $$\begin{array}{cc} (f_{n}\left(t\right)+\epsilon_{0})-f\left(t\right) & \leq 2\epsilon_{0}, \end{array}$$

(A60) $$\begin{array}{cc} \left|\left\lceil f_{n}^{\prime }\left(t\right)-\epsilon_{1}\right\rfloor -f^{\prime }\left(t\right)\right| & \leq 2\epsilon_{1}. \end{array}$$

Hence, in analogy to Theorem 1, the estimation error of ${\underline{I}}_{n}$ can be written as

(A61) $$I\left(f\right)-{\underline{I}}_{n}=\int_{-k_{n}}^{k_{n}}\frac{{\left(f^{\prime }\left(t\right)\right)}^{2}}{f(t)}-\frac{{\left\lceil f_{n}^{\prime }\left(t\right)-\epsilon_{1}\right\rfloor }^{2}}{f_{n}\left(t\right)+\epsilon_{0}}\;dt+c\left(k_{n}\right).$$

Using the same arguments as in the proof of Theorem 1, the integral term on the right-hand side of (A61) can be bounded by

 ∫−knkn(f′(t))2f(t)−⌈fn′(t)−ϵ1⌋2fn(t)+ϵ0dt(A62)= ∫−knkn⌈fn′(t)−ϵ1⌋2f(t)−(f′(t))2(fn(t)+ϵ0)f(t)(fn(t)+ϵ0)dt ≤ ∫−knkn⌈fn′(t)−ϵ1⌋−f′(t)⌈fn′(t)−ϵ1⌋+f′(t)fn(t)+ϵ0dt(A63)+∫−knknf(t)−(fn(t)+ε0)(f′(t))2f(t)(fn(t)+ε0)dt(A64)≤2ϵ1∫−knkn⌈fn′(t)−ϵ1⌋+|f′(t)|fn(t)+ϵ0dt+2ϵ0∫−knkn(f′(t))2f(t)(fn(t)+ϵ0)dt(A65)≤2ϵ1∫−knkn2f′(t)f(t)dt+2ϵ0∫−knknf′(t)f(t)2dt(A66)≤4ϵ1∫−knknρ(t)+2ϵ0∫−knknρ2(t)dt(A67)=4ϵ1Φ1(kn)+2ε0Φ2(kn).

Using the same steps, it is not difficult to show (A51), where the factor 2 does not arise because, in contrast to (A59) and (A60),

(A68) $$\begin{array}{cc} \left\lceil f_{n}\left(t\right)+\epsilon_{0}\right\rfloor -\left\lceil f_{n}\left(t\right)+\gamma_{0,n}\left(t\right)\right\rfloor & \leq \epsilon_{0}, \end{array}$$

(A69) $$\begin{array}{cc} \left\lceil f_{n}^{\prime }\left(t\right)-\gamma_{1,n}\left(t\right)\right\rfloor -\left\lceil f_{n}^{\prime }\left(t\right)-\epsilon_{1}\right\rfloor & \leq \epsilon_{1}, \end{array}$$

and $c(k_{n})$ does not arise because both estimators are defined on $\left[-k_{n},k_{n}\right]$.

In order to show (A49), first note that for $\left|\rho_{n}\left(t\right)\right|\leq \left|\bar{\rho }\left(t\right)\right|$, it holds that

(A70) $$\frac{{\left\lceil f_{n}^{\prime }\left(t\right)-\gamma_{1,n}\left(t\right)\right\rfloor }^{2}}{f_{n}\left(t\right)+\gamma_{0,n}\left(t\right)}=\frac{{\left(f_{n}^{\prime }\left(t\right)\right)}^{2}}{f_{n}\left(t\right)}=\left|\rho_{n}\left(t\right)\right|\left|f_{n}^{\prime }\left(t\right)\right|,$$

i.e., ${\bar{I}}_{n}\left(f_{n}\right)=I_{n}^{c}\left(f_{n}\right)$. Hence, $I_{n}^{c}\left(f_{n}\right)>\bar{I}\left(f_{n}\right)$ implies $|\rho_{n}\left(t\right)|\geq |\bar{\rho }\left(t\right)|$ on some region of $\left[-k_{n},k_{n}\right]$. On this region, it holds that

(A71)⌈fn′(t)−γ1,n(t)⌋2fn(t)+γ0,n(t)=|⌈fn′(t)−γ1,n(t)⌋|fn(t)+γ0,n(t)|⌈fn′(t)−γ1,n(t)⌋|(A72)=|ρ¯(t)||⌈fn′(t)−γ1,n(t)⌋|.

Because

(A73) $$\left|f_{n}^{\prime }\left(t\right)\right|-\left|\left\lceil f_{n}^{\prime }\left(t\right)-\gamma_{1,n}\left(t\right)\right\rfloor \right|\leq \gamma_{1,n}\leq \epsilon_{1},$$

it follows that

(A74)Inc(fn)−I¯n(fn)≤∫−knkn|ρ¯(t)|ϵ1dt(A75)≤ϵ1Φmax1(kn).

## Appendix F. A Proof of Lemma 2

We begin by bounding $v_{r}$ and $\delta_{r,a_{r}}$. First,

(A76) $$\begin{array}{cc} v_{0} & =\int_{-\infty }^{\infty }\left|t\right|K\left(t\right)dt=\sqrt{\frac{2}{\pi }}, \end{array}$$

(A77) $$\begin{array}{cc} v_{1} & =\int_{-\infty }^{\infty }\left|t^{2}-1\right|K\left(t\right)dt=2\sqrt{\frac{2}{e\pi }}. \end{array}$$

Second,

(A78)δr,ar= E[fn(r)(t)]−fY(r)(t)(A79)= ∫−∞∞1arKt−yarfY(r)(y)−fY(r)(t)dy(A80)= ∫−∞∞KyfY(r)(t+ary)−fY(r)(t)dy(A81)≤supt∈RfY(r+1)(t)∫−∞∞Kyar|y|dy(A82)=ar2πsupt∈RfY(r+1)(t).

Now, for r=0,

(A83)fY(1)(t)= E(t−snrX)12πe−(t−snrX)22(A84)≤12π1e,

where we have used the bound $te^{-\frac{t^{2}}{2}}\leq \frac{1}{\sqrt{e}}$. For r=1,

(A85)fY(2)(t)= E(t−snrX)2−112πe−(t−snrX)22(A86)≤12π2e+12π,

where we have used the bound $t^{2}e^{-\frac{t^{2}}{2}}\leq \frac{2}{e}$.

Next, we bound the score function $\rho_{Y}$ as follows:

(A87)|ρY(t)|= fY′(t)fY(t)(A88)= snrE[X|Y=t]−t(A89)≤snrEX|Y=t+|t|(A90)≤snrEX2|Y=t+|t|(A91)≤3snrVar(X)+4t2+|t|(A92)≤3snrVar(X)+3|t|,

where the equality in (A88) follows by using the identify $\frac{f_{Y}^{\prime }\left(t\right)}{f_{Y}\left(t\right)}=\sqrt{\mathrm{snr}}\;E\left[X|Y=t\right]-t$ [23], the inequality in (A90) follows from Jensen’s inequality, and the inequality in (A91) follows from the bound in [24] (Proposition 1.2). Using the bound in (A92), it follows that

(A93) $$\rho_{\max}\left(k_{n}\right)=\max_{\left|t\right|\leq k_{n}}\left|\rho \left(t\right)\right|\leq \sqrt{3\mathrm{snr}\mathrm{Var}(X)}+3k_{n}.$$

Using the relation between the Fisher information and the MMSE, we have that

(A94) $$I(f_{Y})=1-\mathrm{snr}\;\mathrm{mmse}(X,\mathrm{snr})\leq 1.$$

Finally, the function ϕ is obtained by observing that

(A95)fY(t)=E12πe−(t−snrX)22(A96)≥12πe−E(t−snrX)22(A97)≥12πe−t2+snrE[X2],

where we used Jensen’s inequality and the fact that ${\left(a+b\right)}^{2}\leq 2\left(a^{2}+b^{2}\right)$. This concludes the proof.

## Appendix G. A Proof of Lemma 3

Choose some v>0. Then,

(A98)c(kn)=EρY2(Y)1{|Y|≥kn}(A99)≤E11+vρY2(Y)1+vEv1+v1{|Y|≥kn}1+vv(A100)=E11+vρY2(Y)1+vEv1+v1{|Y|≥kn}(A101)=E11+v|ρY(Y)|2(1+v)Pv1+v|Y|≥kn(A102)=E11+v|E[Z|Y]|2(1+v)Pv1+v|Y|≥kn(A103)≤E11+v|Z|2(1+v)Pv1+v|Y|≥kn(A104)=2Γ1(1+v)v+12π12(1+v)Pv1+v|Y|≥kn(A105)=2Γ1(1+v)v+12π12(1+v)snrE[|X|2]+1kn2v1+v,

where (A99) follows from Hölder’s inequality, (A102) follows by using the identity

(A106) $$\rho_{Y}\left(t\right)=\sqrt{\mathrm{snr}}E\left[X|Y=t\right]-t=-E\left[Z|Y=t\right],$$

and (A105) follows from Markov’s inequality.

Now, if $E[X^{2}]<\infty$, then using Markov’s inequality,

(A107) $$P\left[\left|Y\right|\geq k_{n}\right]\leq \frac{E[Y^{2}]}{k_{n}^{2}}=\frac{\mathrm{snr}\;E[|X{|}^{2}]+1}{k_{n}^{2}}.$$

Moreover, using the Chernoff bound,

(A108)P|Y|≥kn≤e−kntEet|Y|(A109)≤2e−knt+t22Eetsnr|X|(A110)=2e−knt+t22eα2snr2.

Therefore,

(A111)c(kn)≤inft>0infv>02Γ1(1+v)v+12π12(1+v)2v1+vev1+v−knt+t22+α2snr2(A112)≤infv>02Γ1(1+v)v+12π12(1+v)2v1+vev1+vα2snr−kn22.

This concludes the proof.

## Appendix H. A Proof of Theorem 4

Let

(A113) $$\varepsilon_{n}=\frac{4\epsilon_{1}k_{n}\rho_{\max}\left(k_{n}\right)+2\epsilon_{1}^{2}k_{n}\phi \left(k_{n}\right)+\epsilon_{0}\phi \left(k_{n}\right)}{1-\epsilon_{0}\phi \left(k_{n}\right)}+c\left(k_{n}\right),$$

which is obtained from (11) by bounding I(f) by 1 according to (31). In order to apply the bounds in Lemma 1 and Theorem 1, the following equalities/inequalities must hold for r∈{0,1}:

(A114a) $$\begin{array}{cc} \epsilon_{r} & >\delta_{r,a_{r}}, \end{array}$$

(A114b) $$\begin{array}{cc} \frac{a_{r}^{2r+2}{\left(\epsilon_{r}-\delta_{r,a_{r}}\right)}^{2}}{v_{r}^{2}} & \gg \frac{1}{n}, \end{array}$$

(A114c) $$\begin{array}{cc} \lim_{n\to \infty }\epsilon_{1}\rho_{\max}\left(k_{n}\right) & =0, \end{array}$$

(A114d) $$\begin{array}{cc} \lim_{n\to \infty }\epsilon_{1}^{2}k_{n}\phi \left(k_{n}\right) & =0, \end{array}$$

(A114e) $$\begin{array}{cc} \lim_{n\to \infty }\epsilon_{0}\phi \left(k_{n}\right) & =0, \end{array}$$

(A114f) $$\begin{array}{cc} \lim_{n\to \infty }c\left(k_{n}\right) & =0. \end{array}$$

To satisfy (A114), we choose

(A115) $$\begin{array}{cc} a_{0}=\epsilon_{0}=n^{-w_{0}} & ,\;w_{0}\in \left(0,\frac{1}{4}\right), \end{array}$$

(A116) $$\begin{array}{cc} a_{1}=\epsilon_{1}=n^{-w_{1}} & ,\;w_{1}\in \left(0,\frac{1}{6}\right), \end{array}$$

(A117) $$\begin{array}{cc} k_{n}=n^{u} & ,\;u\in \left(0,\min\left(w_{0},w_{1}\right)\right). \end{array}$$

Then, together with the bounds in Lemma 2, the relevant quantities in (A114) are as follows:

(A118a) $$\begin{array}{cc} \frac{a_{0}^{2}{\left(\epsilon_{0}-\delta_{0,a_{0}}\right)}^{2}}{v_{0}^{2}} & =\frac{c_{1}}{2}n^{-4w_{0}}, \end{array}$$

(A118b) $$\begin{array}{cc} \frac{a_{1}^{4}{\left(\epsilon_{1}-\delta_{1,a_{1}}\right)}^{2}}{v_{1}^{2}} & =\frac{c_{2}}{2}n^{-6w_{1}}, \end{array}$$

(A118c) $$\begin{array}{cc} \epsilon_{1}k_{n}\rho_{\max}\left(k_{n}\right) & \leq \left(c_{3}\sqrt{u\log(n)}+3u\log\left(n\right)\right)n^{-w_{1}}, \end{array}$$

(A118d) $$\begin{array}{cc} \epsilon_{1}^{2}k_{n}\phi \left(k_{n}\right) & \leq c_{5}\sqrt{u\log(n)}n^{u-2w_{1}}, \end{array}$$

(A118e) $$\begin{array}{cc} \epsilon_{0}\phi \left(k_{n}\right) & \leq c_{5}n^{u-w_{0}}, \end{array}$$

(A118f) $$\begin{array}{cc} c(k_{n}) & \leq \frac{c_{4}}{\sqrt{u\log(n)}}, \end{array}$$

which yields (37). Now, if |X| is α-sub-Gaussian, the bound in (43) can be obtained from Lemma 3 with v=1.

Because (9) leads to (11), one obtains

 PIn(fn)−I(fY)≥εn(A119)≤Psup|t|≤knfn(t)−fY(t)≥ϵ0 + Psup|t|≤knfn′(t)−fY′(t)≥ϵ1(A120)≤Psupt∈Rfn(t)−fY(t)>ϵ0 + Psupt∈Rfn′(t)−fY′(t)>ϵ1(A121)≤2e−nπa02ϵ0−a012πe2+2e−neπa14ϵ1−a12e+12π2(A122)=2e−π1−12πe2n1−4w0+2e−eπ1−2e+12π2n1−6w1(A123)=2e−c1n1−4w0+2e−c2n1−6w1,

where the inequality in (A121) follows from Lemma 1, and the last step follows from (A115), (A116), and (A117). This concludes the proof.

## Appendix I. A Proof of Theorem 5

Let

(A124) $$\varepsilon_{n}=4\epsilon_{1}\Phi_{\max}^{1}\left(k_{n}\right)+2\epsilon_{0}\Phi_{\max}^{2}\left(k_{n}\right)+c\left(k_{n}\right).$$

To apply the bounds in Theorem 3 and Lemma 2, the following equalities/inequalities must hold for r∈{0,1}:

(A125a) $$\begin{array}{cc} \epsilon_{r} & >\delta_{r,a_{r}}, \end{array}$$

(A125b) $$\begin{array}{cc} \frac{a_{r}^{2r+2}{\left(\epsilon_{r}-\delta_{r,a_{r}}\right)}^{2}}{v_{r}^{2}} & \gg \frac{1}{n}, \end{array}$$

(A125c) $$\begin{array}{cc} \lim_{n\to \infty }\epsilon_{1}\Phi_{\max}^{1}\left(k_{n}\right) & =0, \end{array}$$

(A125d) $$\begin{array}{cc} \lim_{n\to \infty }\epsilon_{0}\Phi_{\max}^{0}\left(k_{n}\right) & =0, \end{array}$$

(A125e) $$\begin{array}{cc} \lim_{n\to \infty }c\left(k_{n}\right) & =0. \end{array}$$

To satisfy (A125), we choose

(A126) $$\begin{array}{cc} a_{0}=\epsilon_{0}=n^{-w_{0}} & ,\;w_{0}\in \left(0,\frac{1}{4}\right), \end{array}$$

(A127) $$\begin{array}{cc} a_{1}=\epsilon_{1}=n^{-w_{1}} & ,\;w_{1}\in \left(0,\frac{1}{6}\right), \end{array}$$

(A128) $$\begin{array}{cc} k_{n}=n^{u} & ,\;u\in \left(0,\min\left(\frac{w_{0}}{3},\frac{w_{1}}{2}\right)\right). \end{array}$$

Then, together with the bounds in Lemma 2, the relevant quantities in (A125) are as follows:

(A129a) $$\begin{array}{cc} \frac{a_{0}^{2}{\left(\epsilon_{0}-\delta_{0,a_{0}}\right)}^{2}}{v_{0}^{2}} & =\frac{c_{1}}{2}n^{-4w_{0}}, \end{array}$$

(A129b) $$\begin{array}{cc} \frac{a_{1}^{4}{\left(\epsilon_{1}-\delta_{1,a_{1}}\right)}^{2}}{v_{1}^{2}} & =\frac{c_{2}}{2}n^{-6w_{1}}, \end{array}$$

(A129c) $$\begin{array}{cc} \epsilon_{1}\Phi_{\max}^{1}\left(k_{n}\right) & =n^{u-w_{1}}\left(2c_{3}+3n^{u}\right), \end{array}$$

(A129d) $$\begin{array}{cc} \epsilon_{0}\Phi_{\max}^{1}\left(k_{n}\right) & =n^{u-w_{0}}\left(2c_{3}^{2}+6n^{2u}\right), \end{array}$$

(A129e) $$\begin{array}{cc} c(k_{n}) & \leq c_{4}n^{-u}, \end{array}$$

which yields (46). Moreover, if |X| is α-sub-Gaussian, the bound in (47) can be obtained from Lemma 3.

Following the same steps leading to (A123), we have that

(A130)PInc(fn)−I(fY)≥εn≤2e−π1−12πe2n1−4w+2e−eπ1−2e+12π2n1−6w(A131)=2e−c1n1−4w0+2e−c2n1−6w1.

This concludes the proof.
